# Effect of carbamazepine and gabapentin on excitability in the trigeminal subnucleus caudalis of neonatal rats using a voltage-sensitive dye imaging technique

**DOI:** 10.1186/s40659-015-0027-6

**Published:** 2015-07-21

**Authors:** Akiko Matsumoto, Hirofumi Arisaka, Yuki Hosokawa, Shigeki Sakuraba, Takeo Sugita, Nobuo Umezawa, Yuki Kaku, Kazu-ichi Yoshida, Shun-ichi Kuwana

**Affiliations:** Division of Anesthesiology, Department of Clinical Care Medicine, Kanagawa Dental College, Yokosuka, Kanagawa 238-8580 Japan; Department of Anesthesiology, Kitasato University School of Medicine, Sagamihara, Kanagawa 252-0375 Japan; Center for Medical Sciences, Ibaraki Prefectural University of Health Sciences, Ami, Inashiki-gun, Ibaraki 300-0394 Japan; Faculty of Health Sciences, Uekusa Gakuen University, Ogura-cho, Wakaba-ku, Chiba, 264-0007 Japan

**Keywords:** Carbamazepine, Gabapentin, Rat brainstem, Spinal trigeminal nucleus, Voltage-sensitive dye imaging

## Abstract

**Background:**

The antiepileptic drugs carbamazepine and gabapentin are effective in treating neuropathic pain and trigeminal neuralgia. In the present study, to analyze the effects of carbamazepine and gabapentin on neuronal excitation in the spinal trigeminal subnucleus caudalis (Sp5c) in the medulla oblongata, we recorded temporal changes in nociceptive afferent activity in the Sp5c of trigeminal nerve-attached brainstem slices of neonatal rats using a voltage-sensitive dye imaging technique.

**Results:**

Electrical stimulation of the trigeminal nerve rootlet evoked changes in the fluorescence intensity of dye in the Sp5c. The optical signals were composed of two phases, a fast component with a sharp peak followed by a long-lasting component with a period of more than 500 ms. This evoked excitation was not influenced by administration of carbamazepine (10, 100 and 1,000 μM) or gabapentin (1 and 10 μM), but was increased by administration of 100 μM gabapentin. This evoked excitation was increased further in low Mg^2+^ (0.8 mM) conditions, and this effect of low Mg^2+^ concentration was antagonized by 30 μM DL-2-amino-5-phosphonopentanoic acid (AP5), a *N*-methyl-d-aspartate (NMDA) receptor blocker. The increased excitation in low Mg^2+^ conditions was also antagonized by carbamazepine (1,000 μM) and gabapentin (100 μM).

**Conclusion:**

Carbamazepine and gabapentin did not decrease electrically evoked excitation in the Sp5c in control conditions. Further excitation in low Mg^2+^ conditions was antagonized by the NMDA receptor blocker AP5. Carbamazepine and gabapentin had similar effects to AP5 on evoked excitation in the Sp5c in low Mg^2+^ conditions. Thus, we concluded that carbamazepine and gabapentin may act by blocking NMDA receptors in the Sp5c, which contributes to its anti-hypersensitivity in neuropathic pain.

## Background

The antiepileptic agents carbamazepine and gabapentin are effective against neuropathic pain and trigeminal neuralgia [1, 2]. However, the action of these antiepileptic drugs on neuronal activity in the brain may not be simple. A large body of evidence indicates that carbamazepine may interact with different types of ion channels and synaptic transmission [3, 4]. The molecular targets for ion channels have generally been voltage-gated Na^+^ channels [5], Ca^2+^ channels [6] and K^+^ channels [7]. An increasing number of findings indicate that carbamazepine induces the inhibition of glutamate release [8], inhibition of an adenosine receptor [9] and modulation of neuromodulator levels, such as those of serotonin, dopamin and cyclic adenosine monophosphate (AMP) [3]. In addition, the effects of gabapentin on neural activity are not explained by a single mechanism [10, 11]. Although gabapentin is a structural analogue of γ amino-butyric acid (GABA), it has no affinity for GABA receptors. The main target of gabapentin is synaptic transmission, where it inhibits voltage-gated Ca^2+^ channels in the presynaptic membrane, which inhibits the release of glutamate and substance P [12]. Recent evidence suggests that other actions of gabapentin include inhibition of glutamatergic *N*-methyl-d-aspartate (NMDA) receptors [13] and an increase in GABA release [14].

To study the neural mechanisms of trigeminal neuralgia, trigeminal nerve-attached brainstem preparations from neonatal rats are used [15, 16]. In electrophysiological studies, activity-dependent neuronal hyperexcitability, so-called central sensitization, has been reported in the spinal trigeminal subnucleus caudalis (Sp5c), which receives nociceptive information from the orofacial area. Furthermore, such studies have shown that NMDA receptors contribute substantially to polysynaptic transmission in the Sp5c and to long-term potentiation.

To analyze the spatial dynamics of neuronal excitation propagation in the Sp5c, it is possible to use optical imaging analysis and voltage-sensitive dyes [17, 18]. A previous study using trigeminal nerve-attached brainstem slices from postnatal rats showed that synaptic transmission via unmyelinated afferents in the Sp5c was mediated substantially by NMDA receptors [19]. In the present study, we examined the effects of carbamazepine and gabapentin on excitability in the Sp5c of neonatal rats using an optical imaging technique. Furthermore, we confirmed the contribution of NMDA receptors to enhanced excitability in the Sp5c in low Mg^2+^ concentration conditions.

## Results

### Influence of carbamazepine on evoked excitation in the Sp5c

**Figure 1:**
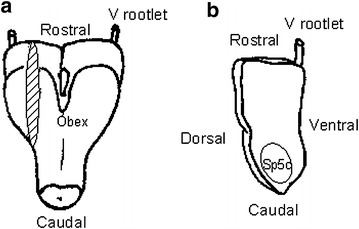
Preparations used for membrane potential imaging. **a** Isolated trigeminal nerve-brainstem preparation. The *dorsal side* is shown at the *front*, and the sagittal sectional surface is shown by hatching. **b** Trigeminal nerve-attached brainstem sagittal slice preparation. The preparation was placed with the sagittal sectional surface upwards for measurement. Electric stimulation was applied by sucking the trigeminal nerve root with a suction electrode for all preparations. *Sp5c* spinal trigeminal subnucleus caudalis, *V rootlet* trigeminal nerve rootlet.

**Figure 2:**
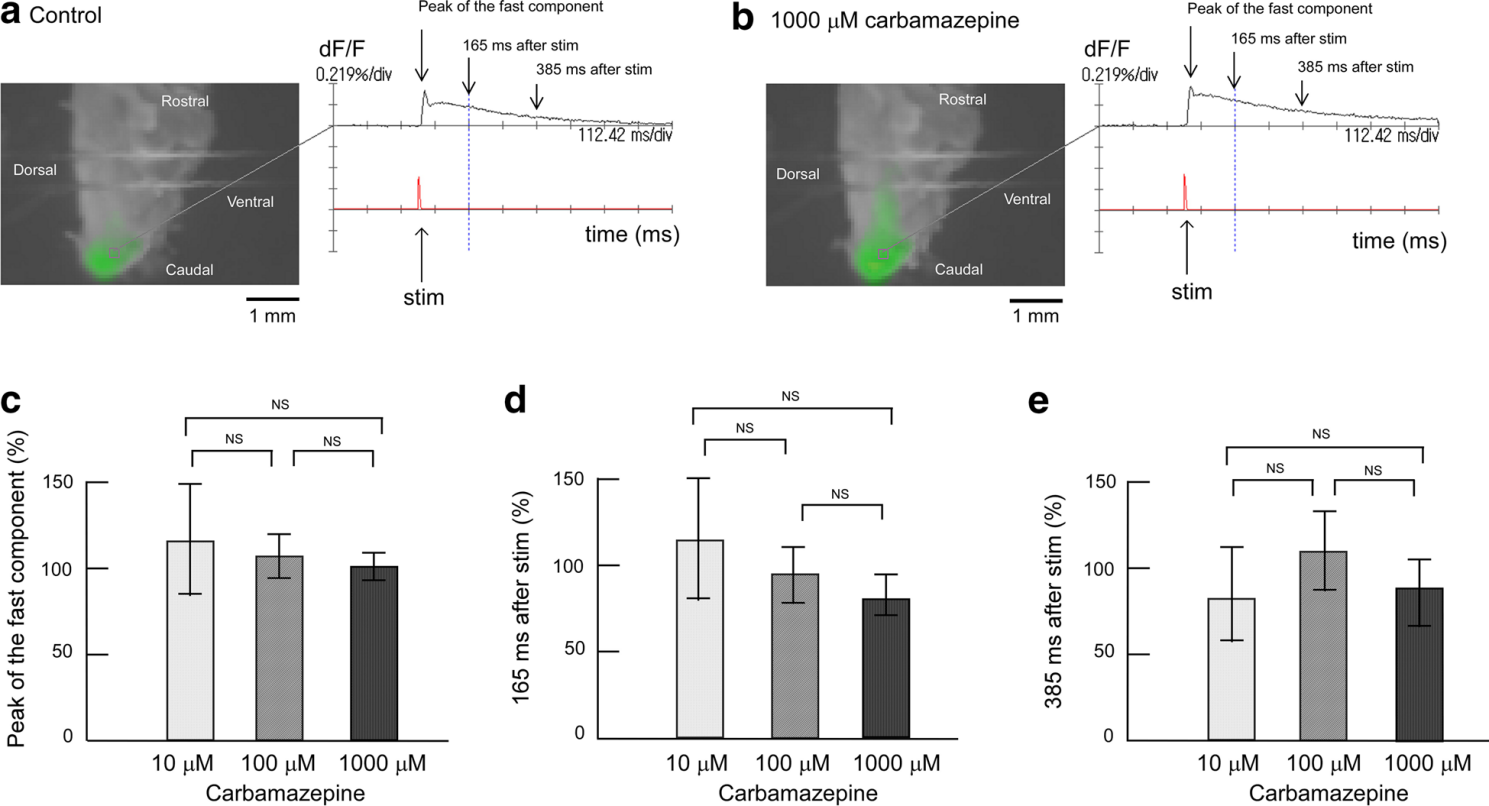
Effect of carbamazepine on evoked excitation in the Sp5c. **a** Recording made during superfusion with control mock CSF. The *left panels* represent neural activity, which is indicated as change in fluorescence intensity using pseudocolor. The *right panel* shows the time course of the signal change and time of electrical stimulation. Optical image in the *left panel* shows 165 ms after stimulation, as indicated by the *vertical dotted line* in the *right panel*. *Two horizontal lines* in the image are the anchors for slice preparation. **b** Recording made during superfusion with mock CSF containing 1,000 μM carbamazepine. **c** Peak amplitudes of evoked excitation in the Sp5c (indicated by *arrows* in the *right panels* of **a**, **b**) were measured during superfusion with 10, 100 and 1,000 μM carbamazepine. **d** Fluorescence signal amplitudes at 165 ms after stimulation (indicated by *arrows* in the *right panels* of **a**, **b**) were measured during superfusion with 10, 100 and 1,000 μM carbamazepine. **e** Fluorescence signal amplitudes at 385 ms after stimulation (indicated by *arrows* in the *right panels* of **a**, **b**) were measured during superfusion with 10, 100 and 1,000 μM carbamazepine. In each graph, fluorescence signal amplitude is indicated as the percent amplitude of the control value during superfusion with control mock CSF. Data of each concentration were obtained from six preparations and are presented as mean ± SD. Carbamazepine did not induce significant changes in evoked excitation in the Sp5c. *NS* not significant.

The influence of drug administration on evoked excitation was examined using trigeminal nerve-attached brainstem sagittal slice preparations, as shown in Figure 1. Figure 2 shows the influence of carbamazepine at different concentrations (10, 100 or 1,000 μM) on evoked excitation in the Sp5c. During superfusion with mock cerebrospinal fluid (CSF) (Figure 2a), the optical signals were composed of two phases, a fast component with a sharp peak followed by a long-lasting component with a period of more than 500 ms. The time delay from stimulation to the peak of the first component was 36.8 ± 8.3 ms (n = 6). The distance from the nerve rootlet to the Sp5c was approximately 4.0 mm. Therefore, the conduction velocity was approximately 0.11 m/s.

The intensity and time course of the optical signal did not alter significantly after switching to superfusion with 1,000 μM carbamazepine-containing mock CSF (Figure 2b). The left-hand panels in Figure 2a, b show fluorescent signal images at 165 ms after electrical stimulation during superfusion with mock CSF and with 1,000 μM carbamazepine-containing mock CSF, respectively. The right-hand panels in Figure 2a, b show time-courses of fluorescent signal changes during superfusion with mock CSF (Figure 2a) and with 1,000 μM carbamazepine-containing CSF (Figure 2b), respectively. When electrical stimulation was applied in the presence of 1,000 μM carbamazepine, the time-course of Sp5c excitement showed a sharp peak after stimulation followed by a slow decline, similar to that in the control. To analyze signal amplitudes during superfusion with 10, 100 and 1,000 μM carbamazepine, fluorescence signal amplitude was indicated as the percent amplitude of the control conditions. The peak amplitude of the first component seemed to decrease slightly to 96.0 ± 12.5% during superfusion of 1,000 μM carbamazepine, but the difference was not significant (Figure 2c). The peak amplitude of the first component was not affected by any concentration of carbamazepine (Figure 2c). To evaluate the effect of carbamazepine on the long-lasting component, we measured the evoked signal at 165 and 385 ms after stimulation (Figure 2d, e), but the signal amplitude of the long-lasting component was also unaffected by carbamazepine (Figure 2d, e).

### Influence of gabapentin on evoked excitation in the Sp5c

**Figure 3:**
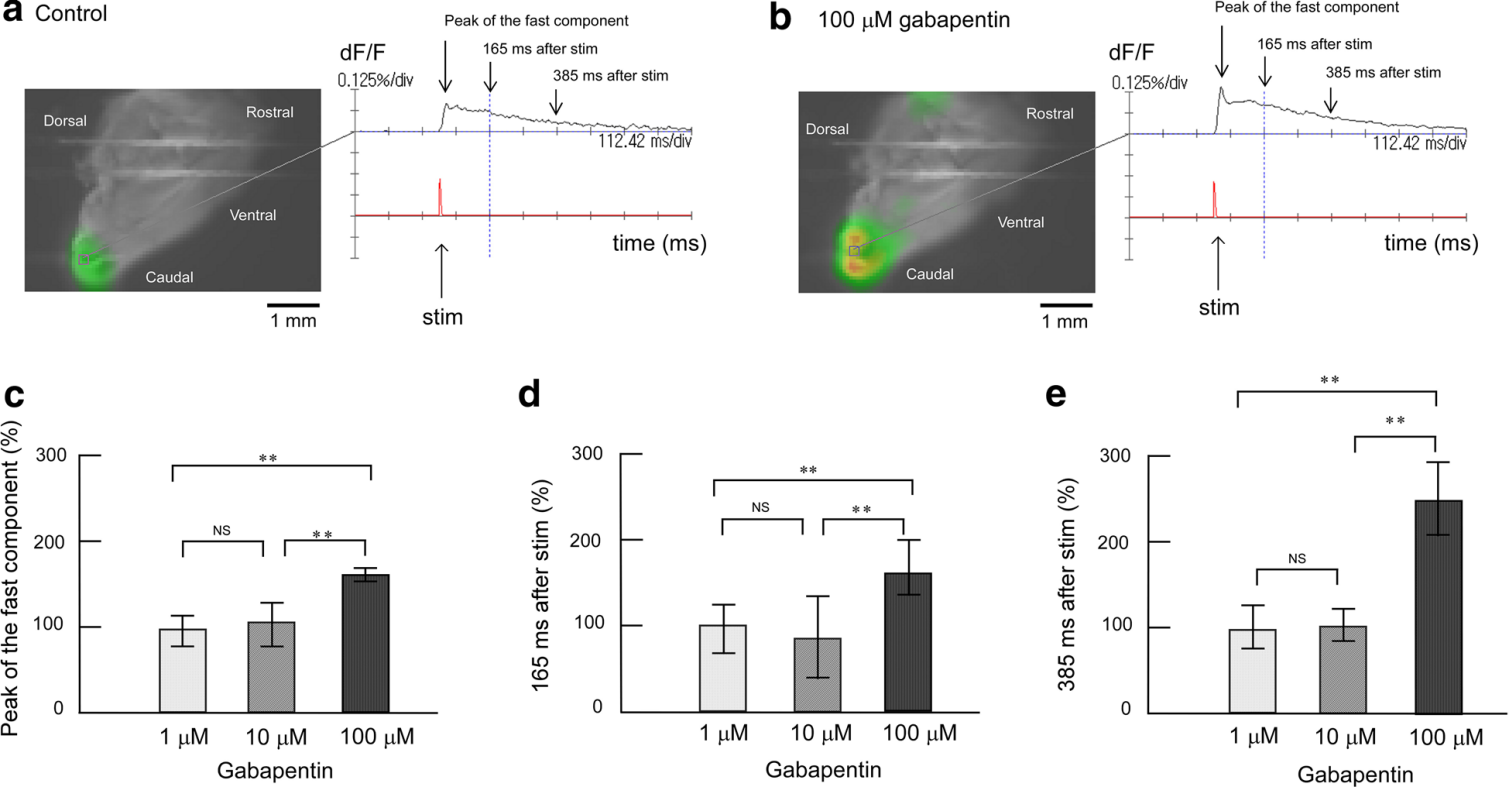
Effect of gabapentin on evoked excitation in the Sp5c. **a** Recording made during superfusion with control mock CSF. The *left panels* represent neural activity, which is indicated as changes in fluorescence intensity using pseudocolor. The *right panel* shows the time course of the signal change and time of electrical stimulation. Optical image in the *left panel* shows 165 ms after stimulation, as indicated by the *vertical dotted line* in the *right panel*. *Two horizontal lines* in the image are the anchors for slice preparation. **b** Recording made during superfusion with mock CSF containing 100 μM gabapentin. **c** Peak amplitudes of evoked excitation in the Sp5c (indicated by *arrows* in the *right panels* of **a**, **b**) were measured during superfusion with 1, 10 and 100 μM gabapentin. **d** Fluorescence signal amplitudes at 165 ms after stimulation (indicated by *arrows* in the *right panels* of **a**, **b**) were measured during superfusion with 1, 10 and 100 μM gabapentin. **e** Fluorescence signal amplitudes at 385 ms after stimulation (indicated by *arrows* in the *right panels* of **a**, **b**) were measured during superfusion with 1, 10 and 100 μM gabapentin. In each graph, fluorescence signal amplitude is indicated as the percent amplitude of the control value during superfusion with control mock CSF. Data of each concentration were obtained from six preparations and are presented as mean ± SD. Administration of 1 and 10 μM gabapentin did not induce significant changes in evoked excitation in the Sp5c, but 100 μM gabapentin emphasized the evoked excitation. **P < 0.01; *NS* not significant.

The dose–response relationship of gabapentin with the evoked excitation was examined using trigeminal nerve-attached brainstem sagittal slice preparations (Figure 3). When electrical stimulation was applied in the presence of 1 or 10 μM gabapentin, the time-course of Sp5c excitement showed a sharp peak after stimulation followed by a slow decline, similar to that in the control conditions. The signal amplitudes of the fast and long-lasting components were not affected by 1 or 10 μM gabapentin (Figure 3c–e). Of note, 100 μM gabapentin significantly increased the peak amplitude of the fast component to 178 ± 5.66% (p < 0.01). Signal amplitudes at 165 and 385 ms after stimulation increased to 179 ± 23.5% (Figure 3d) and 240 ± 31.2%, respectively (Figure 3e). Thus, 100 μM gabapentin induced further excitation in the electrical trigeminal nerve root stimulation-induced excitement of the Sp5c.

### Influence of low Mg^2+^ concentration and an NMDA antagonist on evoked excitation in the Sp5c

**Figure 4:**
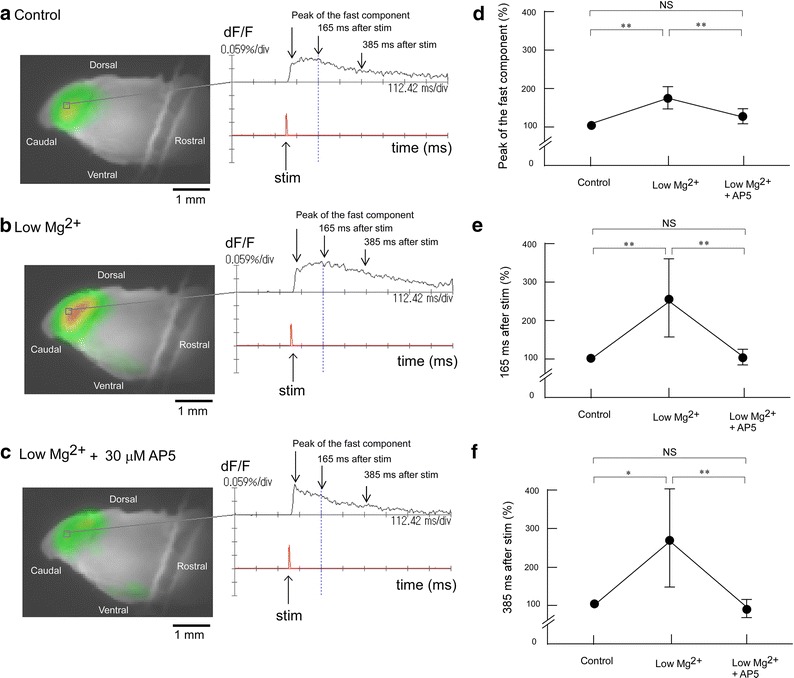
Effect of low Mg^2+^ concentration solution and AP5 on evoked excitation in the Sp5c. **a** Recording made during superfusion with control mock CSF. **b** Recording made during superfusion with low Mg^2+^ concentration (0.8 mM) solution. **c** Recording made during superfusion with low Mg^2+^ solution containing 30 μM AP5. **d** Peak amplitudes of evoked excitation in the Sp5c (indicated by *arrows* in the *right panels* of **a**–**c**) were measured during superfusion with control solution, with low Mg^2+^ solution and with low Mg^2+^ solution containing 30 μM AP5. **e** Fluorescence signal amplitudes at 165 ms after stimulation (indicated by *arrows* in the *right panels* of **a**–**c**) were measured during superfusion with control solution, with low Mg^2+^ solution, and with low Mg^2+^ solution containing 30 μM AP5. **f** Fluorescence signal amplitudes at 385 ms after stimulation (indicated by *arrows* in the *right panels* of **a**–**c**) were measured during superfusion with control solution, with low Mg^2+^ solution, and with low Mg^2+^ solution containing 30 μM AP5. In each graph, fluorescence signal amplitude is indicated as the percent amplitude of the control value during superfusion with control mock CSF. Data were obtained from six preparations and are presented as mean ± SD. Low Mg^2+^ solution induced significant increases in evoked excitation in the Sp5c, but this increase was antagonized by additional administration of 30 μM AP5. *P < 0.05; **P < 0.01; *NS* not significant.

The influence of low Mg^2+^ concentration (0.8 mM) on evoked excitation in the Sp5c was observed using trigeminal nerve-attached brainstem sagittal slice preparations (Figure 4). When electrical stimulation was applied during superfusion with low Mg^2+^ concentration solution, the peak of the fast component after stimulation increased to 177 ± 30.6%. Furthermore, the signal amplitude at 165 and 385 ms after electrical stimulation increased markedly over 250% during superfusion with low Mg^2+^ concentration solution (Figure 4), suggesting that the low Mg^2+^ concentration increased electrical trigeminal nerve root stimulation-induced excitement in the Sp5c. The increase in the long-lasting component with low Mg^2+^ concentration treatment was greater than that of the fast component. A previous study performed by Takuma [19] showed that increased excitation in the Sp5c with low Mg^2+^ concentration treatment was partially antagonized by the NMDA antagonist AP5. We also examined additional superfusion with 30 μM AP5 in low Mg^2+^ conditions. Figure 4c showed that administration of AP5 attenuated the evoked excitement in the Sp5c and restored it to the control level. The peak amplitude was 126 ± 19.2% during superfusion with 30 μM AP5 in low Mg^2+^ conditions (Figure 4d). The amplitude of the long-lasting component was also restored by additional superfusion with 30 μM AP5 in low Mg^2+^ conditions. The amplitudes at 165 and 385 ms after stimulation decreased to 104 ± 21.4% (Figure 4e) and 91.4 ± 31.5% (Figure 4f), respectively.

### Influence of carbamazepine on evoked excitation in the Sp5c in low Mg^2+^ conditions

**Figure 5:**
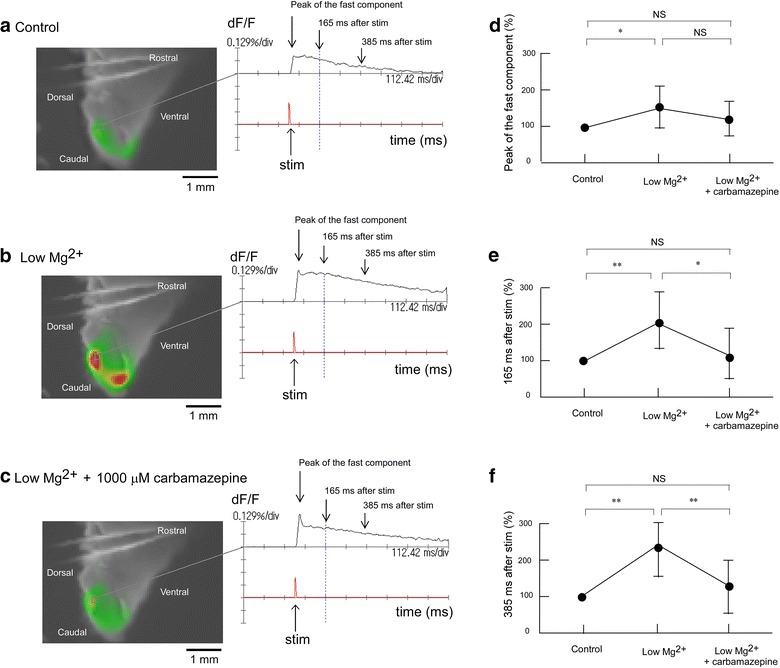
Effect of carbamazepine in low Mg^2+^ conditions on evoked excitation in the Sp5c. **a** Recording made during superfusion with control mock CSF. **b** Recording made during superfusion with low Mg^2+^ concentration (0.8 mM) solution. **c** Recording made during superfusion with low Mg^2+^ solution containing 1,000 μM carbamazepine. **d** Peak amplitudes of evoked excitation in the Sp5c (indicated by *arrows* in the *right panels* of **a**–**c**) were measured during superfusion with control solution, with low Mg^2+^ solution and with low Mg^2+^ solution containing 1,000 μM carbamazepine. **e** Fluorescence signal amplitudes at 165 ms after stimulation (indicated by *arrows* in the *right panels* of **a**–**c**) were measured during superfusion with control solution, with low Mg^2+^ solution, and with low Mg^2+^ solution containing 1,000 μM carbamazepine. **f** Fluorescence signal amplitudes at 385 ms after stimulation (indicated by *arrows* in the *right panels* of **a**–**c**) were measured during superfusion with control solution, with low Mg^2+^ solution and with low Mg^2+^ solution containing 1,000 μM carbamazepine. In each graph, fluorescence signal amplitude is indicated as the percent amplitude of the control value during superfusion with control mock CSF. Data were obtained from six preparations and are presented as mean ± SD. Low Mg^2+^ solution induced significant increases in evoked excitation in the Sp5c, but this increase was antagonized by additional administration of 1,000 μM carbamazepine. *P < 0.05; **P < 0.01; *NS* not significant.

Figure 5 shows the effect of carbamazepine in low Mg^2+^ conditions on evoked excitation in the Sp5c. Superfusion with a low Mg^2+^ concentration solution potentiated the evoked excitation. The effect of these conditions on the long-lasting component (205 ± 67.3% at 165 ms and 237 ± 40.8% at 385 ms after stimulation) was more marked than that on the fast component (162 ± 63.8%). Additional administration of 1,000 μM carbamazepine in low Mg^2+^ conditions attenuated the evoked excitation to the level of the control conditions (Figure 5). The peak amplitude was 130 ± 40.2% during superfusion (Figure 5d). The amplitude of the long-lasting component was also restored by additional superfusion with carbamazepine in low Mg^2+^ conditions. The amplitudes at 165 and 385 ms after stimulation decreased to 113 ± 58.5% (Figure 5e) and 119 ± 60.7% (Figure 5f), respectively. The effect of carbamazepine in low Mg^2+^ conditions was similar to that of AP5.

### Influence of gabapentin on evoked excitation in the Sp5c in low Mg^2+^ conditions

**Figure 6:**
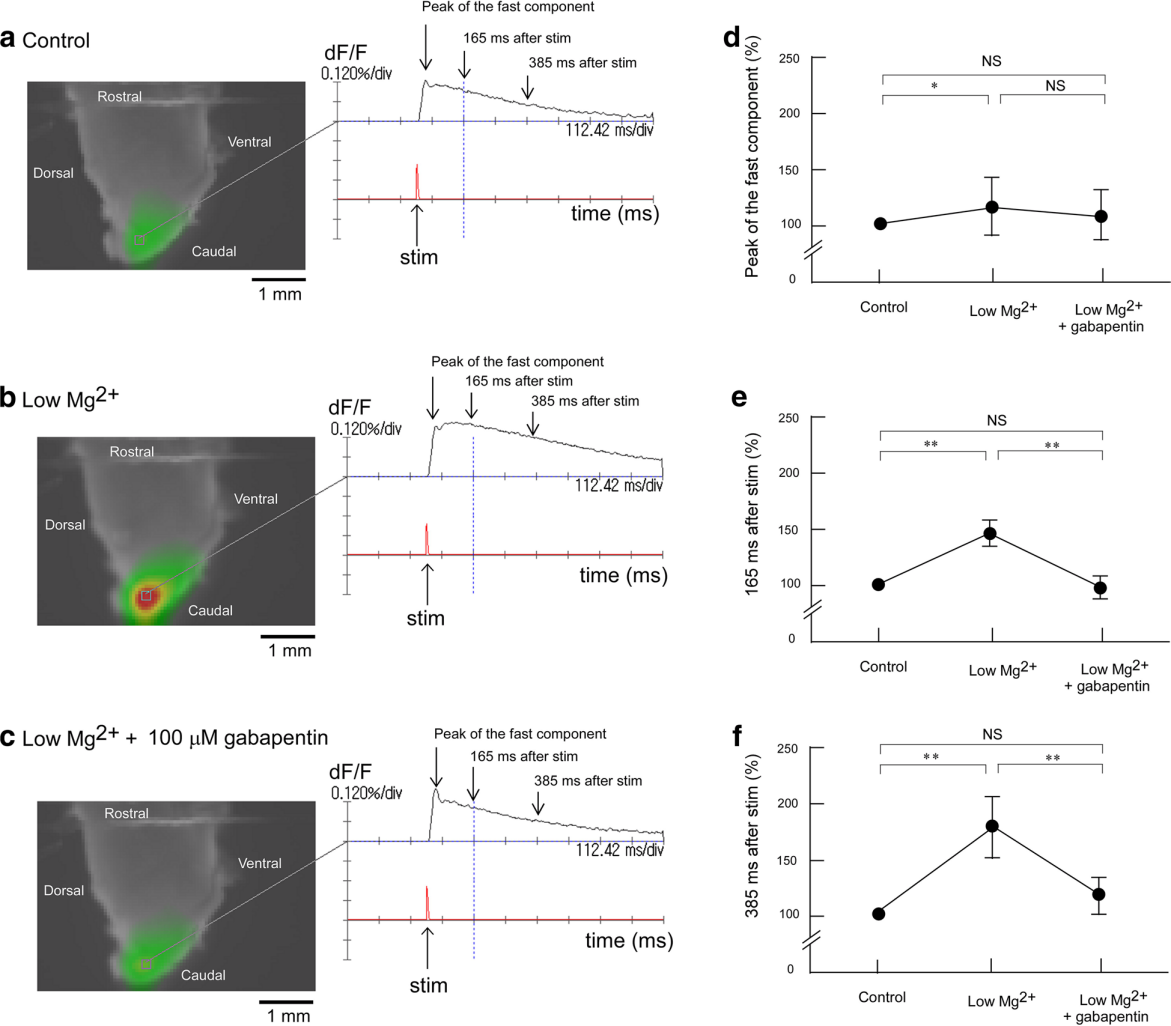
Effect of gabapentin in low Mg^2+^ conditions on evoked excitation in the Sp5c. **a** Recording made during superfusion with control mock CSF. **b** Recording made during superfusion with low Mg^2+^ concentration (0.8 mM) solution. **c** Recording made during superfusion with low Mg^2+^ solution containing 100 μM gabapentin. **d** Peak amplitudes of evoked excitation in the Sp5c (indicated by *arrows* in the *right panels* of **a**–**c**) were measured during superfusion with control solution, with low Mg^2+^ solution and with low Mg^2+^ solution containing 100 μM gabapentin. **e** Fluorescence signal amplitudes at 165 ms after stimulation (indicated by *arrows* in the *right* *panels* of **a**–**c**) were measured during superfusion with control solution, with low Mg^2+^ solution, and with low Mg^2+^ solution containing 100 μM gabapentin. **f** Fluorescence signal amplitudes at 385 ms after stimulation (indicated by *arrows* in the *right panels* of **a**–**c**) were measured during superfusion with control solution, with low Mg^2+^ solution and with low Mg^2+^ solution containing 100 μM gabapentin. In each graph, fluorescence signal amplitude is indicated as the percent amplitude of the control value during superfusion with control mock CSF. Data were obtained from six preparations and are presented as mean ± SD. Low Mg^2+^ solution induced significant increases in evoked excitation in the Sp5c, but this increase was antagonized by additional administration of 100 μM gabapentin. *P < 0.05; **P < 0.01; *NS* not significant.

Figure 6 shows the effect of gabapentin on evoked excitation in the Sp5c in low Mg^2+^ conditions. Superfusion with low Mg^2+^ concentration solution caused potentiation of the evoked excitation. The effect of low Mg^2+^ concentration solution on the long-lasting component (148 ± 14.5% at 165 ms and 186 ± 21.5% at 385 ms after stimulation) was more marked than that on the fast component (128 ± 23.0%). Additional administration of 100 μM gabapentin in low Mg^2+^ conditions attenuated the evoked excitation to the level of the control conditions (Figure 6). The peak amplitude was 119 ± 22.2% during superfusion (Figure 6d). The amplitude of the long-lasting component was also restored by additional superfusion with carbamazepine in low Mg^2+^ conditions. Amplitudes at 165 and 385 ms after stimulation decreased to 97.8 ± 10.7% (Figure 6e) and 115 ± 12.4% (Figure 6f), respectively. The effects of gabapentin in low Mg^2+^ conditions was similar to those of AP5 and carbamazepine.

## Discussion

Applying the voltage-sensitive dye imaging method with isolated neonatal rat brainstem slices allowed electrical trigeminal nerve stimulation-induced excitement of the Sp5c to be temporally and spatially visualized. Sole administration of carbamazepine and of low concentrations of gabapentin did not affect evoked excitation in the Sp5c, although administration of a high concentration of gabapentin emphasized the evoked excitation in the Sp5c. Superfusion with a low Mg^2+^ solution potentiated the evoked excitation. The effect of low Mg^2+^ conditions on the long-lasting component was more marked than that on the fast component. Additional administration of AP5, carbamazepine or gabapentin in low Mg^2+^ conditions attenuated the evoked excitation to the level of the control conditions, showing that the emphasized excitement by low Mg^2+^ concentration was antagonized by AP5, carbamazepine or gabapentin.

Single-pulse stimulation of the trigeminal nerve rootlet induced an optical response in the Sp5c. The optical signals were composed of two phases, a fast component with a sharp peak followed by a long-lasting component with a period of approximately 500 ms. These findings were consistent with those of Takuma [19], who found that the spatiotemporal properties of optical signals were correlated with those of the field potential recordings by using similar preparations to ours. The optical signal of the fast component was eliminated by treatment with 6-cyano-nitro-quinoxaline-2,3-dione (CNQX). The long-lasting component increased in amplitude in low Mg^2+^ conditions but was significantly reduced by AP5. In an electrophysiological study using whole-cell patch-clamp techniques, Onodera et al. [16] reported that stimulation of the mandibular nerve at 0.03 Hz evoked compound excitatory postsynaptic potentials (EPSPs) of neurons in the Sp5c. Compound potentials consistent with monosynaptic EPSPs, which had a high threshold and with polysynaptic EPSPs, was attenuated by high frequency (33–50 Hz) stimulation. In low Mg^2+^ conditions, the fast monosynaptic EPSP component was abolished by simultaneous application of CNQX and AP5, and the slow polysynaptic EPSP was largely attenuated by application of AP5. In the present study, we confirmed the result that AP5 attenuated the fast component and largely attenuated the long-lasting component in low Mg^2+^ conditions. The result that enhanced excitation in low Mg^2+^ conditions was attenuated by application of AP5 suggested that low Mg^2+^ conditions induced the activation of NMDA receptors in the secondary neurons of the Sp5c. Furthermore, we demonstrated that carbamazepine and gabapentin had similar effects to AP5 on evoked excitation in the Sp5c in low Mg^2+^ conditions. This result strongly suggested that carbamazepine and gabapentin act as antagonists of NMDA receptors. Therefore, blockage of NMDA receptors of secondary neurons in the Sp5c may contribute to the clinical effectiveness of carbamazepine and gabapentin.

Inhibition of ion channels and synaptic transmission have been reported as the pharmacological actions of carbamazepine on the nervous system (see “Background”). In control conditions, sole application of carbamazepine did not affect evoked excitement in the Sp5c, suggesting that carbamazepine did not modulate the normal transduction of action potential and synaptic transmission. When excessive activation of the NMDA receptor is induced in the pathophysiology of several neurological conditions, such as trigeminal neuropathic pain, application of carbamazepine was effective because of a block in the activation of NMDA receptors [20, 21].

Regarding the pharmacological action of gabapentin on the nervous system, the inhibition of active potential induction through binding to the α2δ subunit of voltage-dependent Ca^2+^ channels has been reported [22]. Because gabapentin has a high affinity for the α2δ subunit, it was considered to inhibit the release of neurotransmitters by inhibiting Ca^2+^ ion influx [11]. Gabapentin has also been shown to induce the modulation of other targets including NMDA receptors. In the present study, we suggested that the effective site of gabapentin was NMDA receptors in the Sp5c.

One unanticipated finding was that sole application of 100 μM gabapentin emphasized the evoked excitation in control conditions. Petroff et al. [14] reported that gabapentin increased GABA levels in the brains of epileptic patients. Densely packed GABAergic neurons in the Sp5c have been described previously [23, 24]. Because GABAergic transmission in various brain regions of immature animals is excitatory and inhibitory synaptic potentials appear relatively later in development [25], we should consider that not only non-GABAergic but also GABAergic interneurons in this region were excited. It may be possible that a high concentration of gabapentin induced GABA release, and GABA acts as an excitatory transmitter in the Sp5c. However, application of bicuculline further increased the evoked excitation after application of 100 μM gabapentin (unpublished data), suggesting that GABAergic transmission was inhibitory in our preparations. Therefore, the possibility that GABAergic transmission in the Sp5c is excitatory may be ruled out. To clarify the mechanisms of excitation by gabapentin, further studies are required.

In the present study, we estimated the conduction velocity as approximately 0.11 m/s for the peak of the fast component. This value was similar to the value (~0.18 m/s) calculated by previous optical measurements [19]. These values were slow compared with a previous electrophysiological method, in which the conduction velocity was calculated to be 0.37 m/s [16]. Conduction velocities obtained by optical and electrophysiological studies fall into the range of that of C-fibers. On the other hand, trigeminal sensory neurons changed electrophysiological properties during early postnatal maturation. Cabanes et al. [26] showed that trigeminal ganglion neurons in neonatal mice had uniformly slow conduction velocities and separated according to their conduction velocity into Aδ and C neurons during the 3-week postnatal development period. Thus, it is difficult to show which axon type of a trigeminal nerve was mainly stimulated in the present study.

## Conclusion

In conclusion, antiepileptic drugs carbamazepine and gabapentin did not decrease electrically evoked excitation in the Sp5c in control conditions. Further excitation in low Mg^2+^ conditions was attenuated by the NMDA receptor antagonist AP5. Carbamazepine and gabapentin had similar effects to AP5 on evoked excitation in the Sp5c in low Mg^2+^ conditions. Thus, we concluded that carbamazepine and gabapentin act by blocking NMDA receptors in the Sp5c, which contributes to its anti-hypersensitivity action in neuropathic pain and trigeminal neuralgia.

## Methods

### Preparations

All procedures were conducted in accordance with the guidelines of the Uekusa Gakuen University Laboratory Animal Care and Use Committee. Data were obtained from 54 neonatal Wistar rats (2–3 days old). The isolation of brainstem-spinal cord preparations has been described in detail previously [27]. In brief, rats were deeply anesthetized with diethyl ether and the brainstem was isolated in a dissecting chamber at room temperature. The chamber was filled with mock CSF equilibrated with a gas mixture (5% CO_2_ in O_2_; pH 7.4). The composition of the mock CSF was as follows (in mM): NaCl, 126; KCl, 5; CaCl_2_, 2; MgSO_4_, 2; NaH_2_PO_4_, 1.25; NaHCO_3_, 26 and glucose, 30. The cerebrum was quickly removed by transection at the upper border of the inferior colliculus. Each trunk of the bilateral trigeminal nerves that run through the craniobasal bone was isolated to a length of 1 mm, enabling it to be pulled into a suction electrode. Subsequently, the trigeminal nerve-attached brainstem-spinal cord was cut caudally at the level of the C3 roots (Figure 1a). Furthermore, the isolated medulla was sectioned sagittally at 1–1.5 mm lateral from the midsagittal plane with a handmade slicer (Figure 1b). The trigeminal nerve-attached brainstem sagittal slice was placed in a recording chamber (volume 1.0 mL) with the medial side up and continuously superfused (flow 4–6 mL/min) at 26°C with oxygenated mock CSF.

### Voltage-sensitive dye imaging

The voltage-sensitive dye imaging technique has been described in detail previously [28]. In brief, for staining, preparations were kept for 30 min in mock CSF containing the voltage-sensitive dye Di-4-ANEPPS (7.5 mg/mL in 0.1% DMSO, Molecular Probes, Eugene, OR, USA), before being kept for at least 30 min in normal mock CSF. After staining, excess dye was removed by superfusion of the preparation with dye-free solution. After 30 min of washing, optical imaging and data analysis were performed using a MiCAM02 hardware and software package (BrainVision, Tokyo, Japan). For optical imaging, we used a fixed-stage upright fluorescence microscope (Measurescope UM-2, Nikon, Tokyo, Japan) with a low magnification objective lens (XL Fluor 4×/340, Olympus, Tokyo, Japan) and a high-resolution MiCAM02 camera.

To record the voltage-sensitive dye signals, we used light from a 150 W halogen lamp controlled by an electromagnetic shutter (Oriel Instruments, Stratford, USA). Changes in fluorescence of the dye were detected by the camera through a 510–560 nm excitation filter, a dichroic mirror, and a 590 nm absorption filter (MBE1405, Nikon). The camera captured images of 88 × 60 pixels, and the size of the area was 5.4 × 3.7 mm. Optical signals from 3 × 3 pixels (approximately 0.03 mm^2^) were averaged and are showed in the images.

Total frame acquisition was set to 511. Sampling time was 2.2 ms/frame; therefore, the total recording time was 1,124.2 ms. Neuronal activity was evoked by square pulse electrical stimuli (1.0 ms, 0.5–1.0 mA) delivered to the trigeminal nerve rootlet via a glass suction electrode. Acquisition was triggered by the electrical stimulus. The trigger signal was activated after one-quarter of the total recording time, corresponding to 284 ms after starting acquisition when we set the total acquisition time to 1,124.2 ms. Signal amplitude was normalized using the d*F*/*F* method, where *F* is the total fluorescent signal and d*F* corresponds to the change in fluorescence observed following evoked modification of the membrane potential. To improve the signal-to-noise ratio, we averaged signals detected in 10 consecutive trials at 0.3 Hz. To analyze the intensity of the signals, we measured the peak amplitude (at 30–40 ms after stimulation) and amplitudes at 40% (at 165 ms after stimulation) and at 60% (at 385 ms after stimulation) of the total recording time (1,124.2 ms). Measuring points at 165 and 385 ms after stimulation were selected arbitrarily.

### Drug administration

Carbamazepine (Sigma Aldrich, Saint Louis, MO, USA) was added to perfused mock CSF at 10, 100 and 1,000 μM. Gabapentin (Sigma Aldrich) was added to perfused mock CSF at 1.0, 10 and 100 μM. NMDA receptor antagonist DL-2-amino-5-phosphonopentanoic acid (AP5, Sigma Aldrich) was added to perfused mock CSF at 30 μM. These concentrations were determined from preliminary experiments and previous studies [16, 22]. Optical records using electrical stimulation were taken 20 min after the start of superfusion with control mock CSF and were taken 20 min after switching to drug-containing mock CSF. To induce activation of the NMDA receptor, we used low Mg^2+^ concentration solution (in mM): NaCl, 126; KCl, 5; CaCl_2_, 2.6; MgSO_4_, 0.8; NaH_2_PO_4_, 1.25; NaHCO_3_, 26 and glucose, 30. In these series of experiments, optical records using electrical stimulation were taken 20 min after the start of superfusion with control mock CSF and were taken 20 min after switching to low Mg^2+^ concentration solution, and then, taken 20 min after switching to low Mg^2+^ solution containing 30 μM AP5, 1,000 μM carbamazepine or 100 μM gabapentin.

### Data analysis

Optical signal amplitudes obtained before superfusion with mock CSF containing drugs was defined as the control value. The level of statistical significance for the difference between the mean value of each variable obtained during application of different concentrations of drug was conducted by ANOVA followed by pair-wise comparisons using the Tukey–Kramer method for multiple comparisons as indicated. All statistical analyses were conducted using Statcel (OMS publisher, Japan). All values were reported as mean ± SD, and all *P* values < 0.05 were considered significant.
